# Spontaneous voltage oscillations and response dynamics of a Hodgkin-Huxley type model of sensory hair cells

**DOI:** 10.1186/2190-8567-1-11

**Published:** 2011-10-31

**Authors:** Alexander B Neiman, Kai Dierkes, Benjamin Lindner, Lijuan Han, Andrey L Shilnikov

**Affiliations:** 1 Department of Physics and Astronomy, Neuroscience Program, Ohio University, Athens, OH 45701, USA; 2 Max Planck Institute for the Physics of Complex Systems, Nöthnitzer Str. 38, 01187 Dresden, Germany; 3 Bernstein Center for Computational Neuroscience, Physics Department Humboldt University Berlin, Philippstr. 13, Haus 2, 10115 Berlin, Germany; 4 School of Science, Beijing Institute of Technology, 100081 Beijing, People's Republic of China; 5 Neuroscience Institute and Department of Mathematics and Statistics, Georgia State University, Atlanta, GA 30303, USA

## Abstract

We employ a Hodgkin-Huxley-type model of basolateral ionic currents in bullfrog saccular hair cells for studying the genesis of spontaneous voltage oscillations and their role in shaping the response of the hair cell to external mechanical stimuli. Consistent with recent experimental reports, we find that the spontaneous dynamics of the model can be categorized using conductance parameters of calcium-activated potassium, inward rectifier potassium, and mechano-electrical transduction (MET) ionic currents. The model is demonstrated for exhibiting a broad spectrum of autonomous rhythmic activity, including periodic and quasi-periodic oscillations with two independent frequencies as well as various regular and chaotic bursting patterns. Complex patterns of spontaneous oscillations in the model emerge at small values of the conductance of Ca^2+^-activated potassium currents. These patterns are significantly affected by thermal fluctuations of the MET current. We show that self-sustained regular voltage oscillations lead to enhanced and sharply tuned sensitivity of the hair cell to weak mechanical periodic stimuli. While regimes of chaotic oscillations are argued to result in poor tuning to sinusoidal driving, chaotically oscillating cells do provide a high sensitivity to low-frequency variations of external stimuli.

## Introduction

Perception of sensory stimuli in auditory and vestibular organs relies on active mechanisms at work in the living organism. Manifestations of this active process are high sensitivity and frequency selectivity with respect to weak stimuli, nonlinear compression of stimuli with larger amplitudes, and spontaneous otoacoustic emissions [1]. From a nonlinear dynamics point of view, all these features are consistent with the operation of nonlinear oscillators within the inner ear [2,3]. The biophysical implementations of these oscillators remain an important topic of hearing research [1,4-6].

Several kinds of oscillatory behavior have experimentally been observed in hair cells, which constitute the essential element of the mechano-electrical transduction (MET) process. In hair cells, external mechanical stimuli acting on the mechano-sensory organelle, the hair bundle, are transformed into depolarizing potassium currents through mechanically gated ion channels (MET channels). This current influences the dynamics of the basolateral membrane potential of the hair cell and may thus trigger the release of neurotransmitter. In this way, information about the sensory input is conveyed to afferent neurons connected to the hair cell.

Self-sustained oscillations in hair cells occur on two very different levels. First, the mechano-sensory hair bundle itself can undergo spontaneous oscillations and exhibit precursors of the above-mentioned hallmarks of the active process in response to mechanical stimuli [5,7-9]. Second, self-sustained electric voltage oscillations across the membrane of the hair cell have been found. This study is concerned with the second phenomenon, the electrical oscillations.

It has been known for a long time that the electrical compartment of hair cells from various lower vertebrate species, e.g., birds, lizards, and frogs, exhibits damped oscillations in response to step current injections. This electrical resonance has been suggested as a contributing factor to frequency tuning in some inner ear organs [10-13]. Besides these passive oscillations, recent experimental studies in isolated [14,15] and non-isolated [16] saccular hair cells have documented spontaneous self-sustained voltage oscillations associated with Ca^2+ ^and K^+ ^currents. In particular, various regimes of spontaneous rhythmical activity were observed, including small-amplitude oscillations, large-amplitude spikes as well as bursting behavior [16].

Catacuzzeno et al. [14] and Jorgensen and Kroese [15] developed a computational model within the Hodgkin-Huxley formalism that in numerical simulations was shown to reproduce principle features derived from experimental data.

We note that the spontaneous voltage oscillations reported in [14,16] arose solely because of the interplay of basolateral ionic currents and were not caused by an oscillatory MET current associated with hair bundle oscillations. However, *in vivo*, fluctuations of the MET current are expected to severely affect spontaneous voltage oscillations in hair cells, a situation that has not been examined so far. Furthermore, variations of the membrane potential may affect hair bundle dynamics through the phenomenon of reverse electro-mechanical transduction [17,18]. Recent theoretical studies in which voltage oscillations were modeled by a normal form of the Andronov-Hopf (AH) bifurcation [19] or by a linear damped oscillator [20] have shown that the coupled mechanical and electrical oscillators may result in enhanced sensitivity and sharper frequency responses. However, the dynamics of the membrane potential appeared to be far more complicated than mere damped or limit cycle oscillations even in the absence of oscillatory hair bundles [16].

In this article, we study the dynamical properties of the hair cell model proposed in [14] including quiescence, tonic, and bursting oscillations, a quasi-periodic behavior, as well as onset of chaos, and identify the bifurcations underlying the transition between these activity types. To examine the influence of inevitable fluctuations on these dynamical regimes, we extend the model by including a stochastic transduction current originating in the Brownian motion of the hair bundle and channel noise because of the finite number of MET channels [21].

To minimize the number of control parameters and to make results more tractable, we restrict ourselves to a passive model of MET [13] neglecting mechanical adaptation and possible electro-mechanical feedback, leaving consideration of a comprehensive two-compartmental model for a future study.

We show that a small parameter window of chaotic behaviors in the deterministic model can considerably be widened by noise. Furthermore, we discuss the response of the voltage compartment to two kinds of sensory mechanical stimulation of the hair bundle, namely, static and periodic. We find that high sensitivity to static stimuli is positively correlated with the occurrence of chaos in the noisy system (large positive Lyapunov exponent, LE), whereas the maximal sensitivity at finite frequency is achieved for regular oscillations (LE is close to 0 but negative). We discuss possible implications of our findings for the signal detection by hair cells.

## Materials and methods

**Figure 1:**
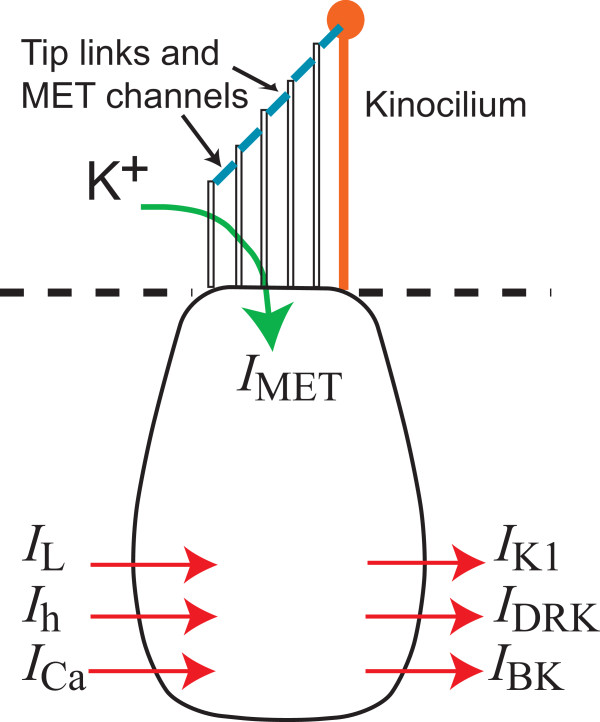
**MET and ionic currents in the hair cell**. Each hair cell is equipped with a mechano-sensory hair bundle, i.e., a tuft of stereocilia that emanates from the apical surface of the cell. Stereocilia are arranged in rows of increasing height, with neighboring stereocilia being interlinked by fine filaments, the so-called tip links. The hair bundle is immersed in K^+^-rich endolymph. In contrast, the basolateral membrane of the hair cell is in contact with perilymph, which is characterized by a low K^+ ^and a high Na^2+ ^ion concentration. Upon deflection of the hair bundle toward the largest row of stereocilia, tension in the tip links increases. This elicits the opening of mechanically gated ion channels (MET channels) that are located near the tips of stereocilia. As a result, K^+ ^ions rush into the hair cell, giving rise to an inward MET current (*I*_MET_, green arrow). The basolateral membrane of the hair cell comprises several types of ion channels, associated with specific ionic currents. Shown are DRK K^+ ^(*I*_DRK_), inwardly rectifier (*I*_K1_), K^+^/Na^2+ ^h-type current (*I*_h_), Ca^2+ ^(*I*_Ca_), and Ca^2+^-activated K^+ ^BK currents (consisting of the steady *I*_BKS _and transient *I*_BKT _currents). Red arrows indicate the directions of ionic currents.

Figure 1 shows a sketch of basolateral ionic currents used in the model analyzed here. The outward potassium currents are as follows: the delayed rectifier (DRK) current, *I*_DRK_; the calcium-activated steady (BKS) and transient (BKT) currents, *I*_BKS _and *I*_BKT_; the inwardly rectifier potassium (K1) current, *I*_K1_. The inward currents are the cation h-type current, *I*_h_; a voltage-gated calcium current, *I*_Ca_; and a leak current, *I*_L_. Two ionic currents, *I*_K1 _and *I*_h_, are activated by hyperpolarization. A fast inactivating outward potassium current (A-type) was not included, as it had negligible effect on the dynamics of the membrane potential [16].

The equation for the membrane potential *V *reads as follows:

(1) $$C_{\text{m}}\frac{dV}{dt}=-I_{\text{K}1}-I_{\text{h}}-I_{\text{DRK}}-I_{\text{Ca}}-I_{\text{BKS}}-I_{\text{BKT}}-I_{\text{L}}-I_{\text{MET}},$$

where *C*_m _= 10 pF is the cell capacitance.

Note that in Equation 1, we also included the inward MET potassium current, *I*_MET_, given by

(2) $$I_{\text{MET}}=g_{\text{MET}}P_{o}\left(X\right)\;\left(V-E_{\text{MET}}\right),$$

where *g*_MET _is the maximum conductance of the MET channels and *P_o_*(*X*) is the open probability of MET channel with a 0 reversal potential, *E*_MET _= 0 mV. For a hair bundle with *N *= 50 transduction channels, we use *g*_MET _= 0.65 nS, which is consistent with measurements according to Holton and Hudspeth [22]. The open probability of the MET channels depends on the displacement of the hair bundle from its equilibrium position. Here, we use a two-state model for the MET channel [22], with the Boltzmann dependence for *P_o_*(*X*) given by

(3) $$P_{o}\left(X\right)=\frac{1}{1+\exp\left[-\frac{Z\left(X-X_{0}\right)}{k_{\text{B}}T}\right]},$$

where *Z *is the gating force, and *X*_0 _is the position of the bundle corresponding to *P_o _*= 0.5. For the sacculus of the bullfrog, the typical values are *Z *= 0.7 pN and *X*_0 _= 12 nm [23]. Thermal fluctuations of the MET current are the main source of randomness in hair cells [18] and stem from the Brownian motion of the hair bundle and random clattering of MET channels (the so-called channel noise). We model the hair bundle as a passive elastic structure with an effective stiffness *K*, immersed in a fluid. Fluid and MET channels result in an effective friction *λ *[21], so that the overdamped stochastic dynamics of the hair bundle is described by the following Langevin equation,

(4) $$\lambda \frac{dX}{dt}=-K\;x+F_{\text{ext}}\left(t\right)+\varepsilon \;\sqrt{2\lambda k_{\text{B}}T}\xi \left(t\right),$$

where *F*_ext _(*t*) is an external stimulating force and *ξ *(*t*) is white Gaussian noise with autocorrelation function 〈*ξ *(*t*) *ξ *(*t *+ *τ*)〉 = *δ*(*τ*). Purely deterministic dynamics correspond to *ε = *0. The numerical values for the other parameters are *λ *= 2.8 *μ*N·s/m [21] and *K *= 1350 *μ*N/m. In the absence of a stimulus, the stochastic dynamics of the hair bundle results in fluctuations of the open probability (3) and consequently of the MET current (2) and serves as the only source of randomness in the model. Indeed, such a model is a severe simplification of hair bundle dynamics as it neglects the adaptation because of myosin molecular motors and the forces which the MET channels may exert on the bundle, i.e., the so-called gating compliance [5]. The equations for the ionic currents, their activation kinetics, and the parameter values used are presented in the Appendix. The model is a system of 12 nonlinear coupled differential equations: one describing the membrane potential *V *(1), two equations for the *I*_K1_, one per *I*_h_, *I*_DRK_, and per *I*_Ca_; 6 equations for the BK currents *I*_BKS _and *I*_BKT_; 1 equation for the calcium dynamics. In addition, Equation 4 describes the stochastic dynamics of a passive hair bundle.

Integration of the model equations was done using the explicit Euler method with the constant time step of 10*^-^*^2 ^ms. A further decrease in the time step did not lead to significant quantitative changes in the dynamics of the system. The bifurcation analysis of the deterministic model was conducted using the software packages CONTENT and MATCONT [24,25] which allow for parameter continuation of equilibrium states and periodic orbits of autonomous systems of ODEs. The largest LE was computed by averaging a divergence rate of two solutions of the model over a long trajectory as follows [26]. We started two trajectories **y**_1_(*t*) and **y**_2_(*t*) in the 12-dimensional phase space of the model subjected to the same realization of noise, but with the initial conditions separated by an initial vector with the norm *d*_0 _= *a*|**y**_1_(0)|, *a *≪ 1. We continued these trajectories for a time interval *τ *= 0.5 - 1 s and calculated the new separation distance between trajectories, *d_m _*= |**y**_2 _(*mτ*) - **y**_1 _(*mτ*)|. The initial conditions of both trajectories were updated to their new values and the norm of the initial vector was normalized back to *d*_0_. This procedure was repeated for *m *= 1,..., *M *iterations until an estimate of the largest LE,

(5) $$\Lambda =\frac{1}{M\tau }\overset{M}{\underset{m=1}{\sum }}\log\left(\frac{d_{m}}{d_{0}}\right),$$

converged.

The power spectral density (PSD) of the membrane potential defined as $G_{VV}\left(f\right)=\left\langle Ṽ\left(f\right)\;{Ṽ}^{*}\left(f\right)\right\rangle$, where Ṽ(f) is the Fourier transform of *V *(*t*), was calculated from long (600 s) time series using the Welch periodogram method with Hamming window [27].

We used an external harmonic force, *F*_ext_(*t*) = *F*_0 _cos(2*πf*_s_*t*), with the amplitude *F*_0 _and the frequency *f*_s _to compute the sensitivity of the hair cell and its dependence on the amplitude and frequency of the external force. The time-dependent average of the membrane potential, 〈*V *(*t*)〉, was estimated by averaging over 200 realizations of *V *(*t*) (length corresponded to 1000 cycles of the driving signal) and the sensitivity was calculated as

(6) $$\chi \left(f_{s}\right)=\frac{\left|{Ṽ}_{\text{mean}}\left(f_{s}\right)\right|}{F_{0}},$$

where ${Ṽ}_{\text{mean}}\left(f_{s}\right)$ is the first Fourier harmonic of 〈*V *(*t*)〉 at the frequency of the external force.

In the regime of linear responses, i.e., for weak stimulation, we used an alternative method of sensitivity estimation [28]: the external force was zero mean broadband Gaussian noise with the standard deviation *σ_s_*, band-limited to the cutoff frequency of *f*_c _= 200 Hz, *F*_ext_(*t*) = *s*(*t*). The PSD of the stimulus was $G_{\text{ss}}\left(f\right)=\sigma_{s}^{2}∕\left(2f_{\text{c}}\right)$ for *f *in [0 *f*_c_] and 0 otherwise. The frequency dependence of the sensitivity was computed as

(7) $$\chi \left(f\right)=\frac{\left|G_{sV}\left(f\right)\right|}{G_{\text{ss}}\left(f\right)},$$

where *G_sV _*is the cross-spectral density between the stimulus, *s*(*t*), and the response *V *(*t*) [29].

This procedure allowed to obtain a frequency tuning curve at once for a given parameter setting, avoiding variation of the frequency of a sinusoidal force. Both sinusoidal and broadband stimuli gave almost identical tuning curves for small stimulus magnitudes *F*_0_, *σ_s _*≤ 1 pN.

### Deterministic dynamics

In the autonomous deterministic case, *ε *= 0 and *F*_ext _= 0 in Equation 4. The hair bundle displacement is *X *= 0 and the open probability of the MET channel is *P_o _*= 0.114, so that the MET current can be replaced by a leak current with the effective leak conductance *g*_L _+ *g*_MET _*P_o _*= 0.174 nS.

### Choice of control parameters

Saccular hair cells in bullfrog are known to be heterogeneous in their membrane potential dynamics, i.e., while some cells exhibit spontaneous tonic and spiking oscillations, others are quiescent [14,16]. Although all bullfrog saccular hair cells possess similar components of the ion current (Figure 1), oscillatory and non-oscillatory cells are characterized by different ratios of specific ion channels involved (see Figure five in [16]). For example, quiescent cells are less prone to depolarization because of a smaller fraction of inward rectifier current (K1) and a larger fraction of outward currents (BK and DRK). Spiking cells, on the contrary, exhibit a larger fraction of K1 and a smaller fraction of BK currents. The importance of BK currents in setting the dynamic regime of a hair cell is further highlighted by the fact that cells can be turned from quiescent to spiking by blocking BK channels [14-16]. In contrast, other currents have similar fractions in oscillatory and non-oscillatory cells, e.g., the cation h-current and the Ca current [16]. Based on these experimental findings, we minimized the number of parameters choosing *b *and *g*_K1_, which determine the strengths of the BK and K1 currents, respectively, as the main control parameters of the model.

### Bifurcations of equilibria and periodic solutions

**Figure 2:**
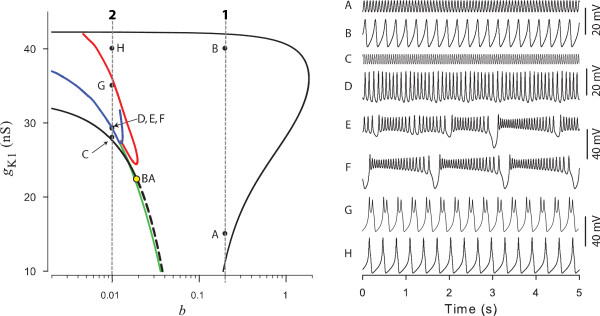
**Dynamical regimes of the deterministic model (*ε *= 0)**. Left: Bifurcation diagram of the model in the (*b*, *g*_K1_)-parameter plane. Black solid and dashed curves correspond (resp.) to supercritical and subcritical AH bifurcations of the depolarized (left branch) and hyperpolarized (right branch) equilibrium state. Open circle indicates the Bautin bifurcation (BA). Green line corresponds to a saddle-node bifurcation of limit cycles. Blue curve indicates a torus birth bifurcation of a stable small-amplitude limit cycle. Red line indicates a period doubling bifurcation of a stable large-amplitude spiking limit cycle. Points labeled A-H correspond to the voltage traces on the right panel: *b *= 0.2, *g*_K1 _= 15 nS (A); *b *= 0.2, *g*_K1 _= 40 nS (B); *b *= 0.01, *g*_K1 _= 28 nS (C); *b *= 0.01, *g*_K1 _= 29.192 nS (D); *b *= 0.01, *g*_K1 _= 29.213 nS (E); *b *= 0.01, *g*_K1 _= 29.25 nS (F); *b *= 0.01, *g*_K1 _= 35 nS (G); *b *= 0.01, *g*_K1 _= 40 nS (H). Other parameters are *g*_L _= 0.174 nS, *g*_MET _= 0, *g*_h _= 2.2 nS, *ε *= 0.

A bifurcation diagram of the model is shown in the left panel of Figure 2. An interior region of oscillatory behavior is separated from a region corresponding to a stable equilibrium (or quiescent) state of the hair cell model by AH bifurcation lines (shown as solid and dashed black lines). The type of the AH bifurcation is determined by the sign of the so-called first Lyapunov coefficient. The supercritical (solid black line) and subcritical (dashed black line) branches of the AH bifurcation are divided by a codimension-two Bautin bifurcation (yellow circle labeled BA in Figure 2, left), at which the first Lyapunov coefficient vanishes. A bifurcation curve of saddle-node periodic orbits (green line, Figure 2, left) originating from the Bautin bifurcation together with the subcritical AH bifurcation curve (dashed black line) singles out a bistability window in the bifurcation diagram of the model. In this narrow region bounded by the saddle-node and the subcritical AH bifurcation curves, the model can produce periodic oscillations or be at equilibrium, depending on the initial conditions.

For relatively large values of *b *(*>*0.02), the model robustly exhibits periodic oscillations or quiescence. For example, if one fixes a value of *b *at 0.2 (dashed grey vertical line **1**, Figure 2, left) then the increase of *g*_K1 _leads to the birth of a limit cycle from the equilibrium state, when crossing the AH curve at *g*_K1 _= 11.4 nS. Further increase of *g*_K1 _does not lead to bifurcations of the limit cycle until *g*_K1 _crosses the AH curve at *g*_K1 _= 42 nS, when the limit cycle bifurcates to a stable hyperpolarized equilibrium state. Smaller values of *b *may result in a sequence of local and non-local bifurcations of periodic orbits. For example, if one fixes *b *at 0.01 and increases *g*_K1 _(grey dashed vertical line **2**, Figure 2, left) then a limit cycle born through the supercritical AH bifurcation at *g*_K1 _= 27.7 nS bifurcates to a torus when *g*_K1 _crosses the torus birth bifurcation curve (blue line, Figure 2, left) at *g*_K1 _≈ 29.2 nS. Further increase of *g*_K1 _results in the destruction of the torus and a cascade of transitions to bursting oscillations (discussed below), until *g*_K1 _reaches a period doubling bifurcation curve (red line, Figure 2, left) at *g*_K1 _≈ 35.6 nS. Crossing the period doubling curve results in a single-period limit cycle oscillation which bifurcates to the hyperpolarized equilibrium state at *g*_K1 _= 42.2 nS.

The right panel of Figure 2 depicts a few typical patterns of spontaneous oscillations of the membrane potential. For *b >*0.02 the model is either equilibrium (quiescence) or exhibits tonic periodic oscillations. Increasing the value of *g*_K1 _leads to hyperpolarization of the cell accompanied with larger amplitude, lower frequency oscillations (Figure 2, points A and B be in the left panel, traces A and B in the right panel). For smaller values of the BK conductance (*b <*0.02), the dynamics of the model is characterized by diverse patterns of various tonic and bursting oscillations as exemplified by points and traces C-E in Figure 2 for the fixed *b *= 0.01. With the increase of *g*_K1 _small-amplitude periodic oscillations (Figure 2C) evolve into quasi-periodic oscillations with two independent frequencies (Figure 2D) via a torus birth bifurcation. In the phase space of the model, the quasi-periodic oscillations correspond to the emergence of a two-dimensional (2D) invariant torus. The quasi-periodic oscillations, occurring within a narrow parameter window, transform abruptly into chaotic large-amplitude bursting shown in Figure 2E. A further increase of *g*_K1 _leads to the regularization of the bursting oscillations with a progressively decreasing number of spikes per burst (Figure 2F, G). Ultimately, a regime of large amplitude periodic spiking is reached (Figure 2H).

**Figure 3:**
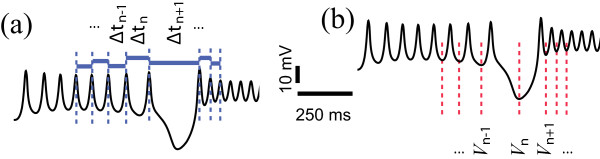
**Bursting voltage trace with varying interspike intervals, Δ*t_n _***(a) **and a sequence of consecutive minima of the membrane potential, *V_n _***(b)****.

Next we extend the analysis of oscillatory behaviors of the hair cell model by employing the effective technique of Poincaré maps developed for describing nonlocal bifurcations of oscillatory dynamics [30-33]. We construct 1D recurrence maps for the instantaneous interspike intervals Δ*t_n _*(see Figure 3a) and consecutive minima, *V_n_*, of the membrane potential (Figure 3b).

**Figure 4:**
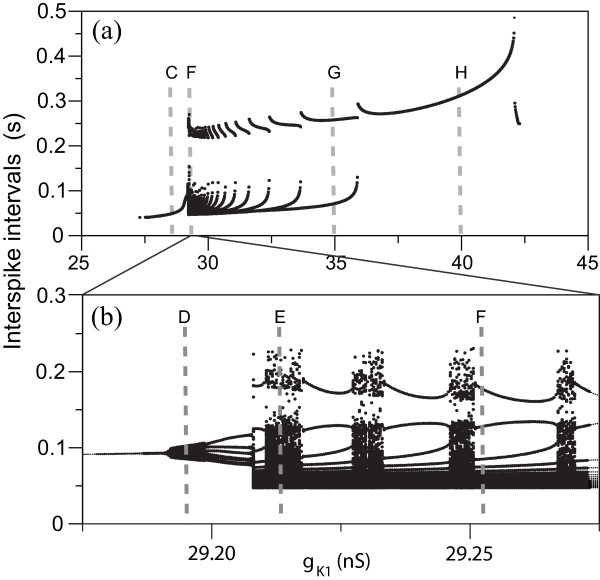
**Bifurcations of oscillatory dynamics in the deterministic model**. **(a) **Bifurcation diagram of the model at *b *= 0.01 for the interspike intervals plotted versus *g*_K1_. **(b) **Zoom into the bifurcation diagram shown in panel **(a)**. Vertical grey dashed lines labeled C-H in **(a) **and **(b) **refer to corresponding points and voltage traces in Figure 2. Other parameters are the same as in Figure 2.

A one-parameter bifurcation diagram^a ^in Figure 4a demonstrates how the interspike intervals evolve as *g*_K1 _varies at a fixed value of the BK conductance, *b *= 0.01. Depending on whether the voltage shows simple or more complex spiking, one observes that the interspike interval attains only one value or several different values, respectively. More specifically, starting at large values *g*_K1 _= 42 nS, one observes that as *g*_K1 _decreases, large-amplitude tonic spiking oscillations (see the corresponding voltage trace in Figure 2H) transform into bursting oscillations by adding initially an extra spike into each burst (Figure 2G). This is reflected in the bifurcation diagram, Figure 4a, as the appearance of short intervals between spikes inside a burst and long intervals between bursts. A further decrease of *g*_K1 _reveals a spike-adding sequence within bursting with variable numbers of spikes. The sequence accumulates to a critical value of *g*_K1 _beyond which the model exhibits small-amplitude tonic oscillations. A zoom of the bifurcation diagram in Figure 4b reveals that each subsequent spike-adding sequence is accompanied by chaotic bursting within a narrow parameter window, in a manner similar to neuronal models [30,31,34-36]. Near the terminal point of the spike-adding cascade, the model generates unpredictably long bursting trains with chaotically alternating numbers of spikes (Figure 2E). So, for small values of the BK conductance, the dynamical source of instability in the model is rooted in homoclinic bifurcations of a saddle equilibrium state, which suggests explicitly that the given model falls into a category of the so-called square-wave bursters introduced for 3D neuronal models [37,38].

### Torus breakdown for bursting

**Figure 5:**
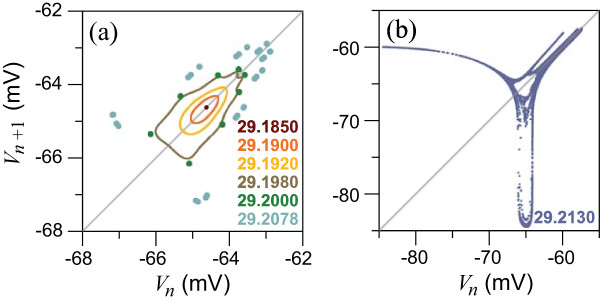
**Recurrence maps for the consecutive minima of the membrane potential for the indicated values of the parameter *g*_K1_**. **(b) **Recurrence map for *g*_K1 _= 29.213 nS (corresponding to chaotic bursting oscillations shown in Figure 2E) emerging via the breakdown of the torus shown in **(a)**. Note the distinct scales used in **(a) **and **(b) **(the original torus would be situated in the middle section of the map shown in **(b)**). Other parameters are the same as in Figure 2.

There is a novel dynamic feature that makes the hair cell model stand out in the list of conventional models of bursting. Namely, at the very end of the spike-adding sequence there is a parameter window where the model generates quasi-periodic oscillations with two independent frequencies (Figure 2D). Such oscillations are associated with the onset (and further breakdown) of a 2D invariant torus in the phase space. A comprehensive study of the torus bifurcations is beyond the scope of this article. Here, we briefly demonstrate some evolutionary stages of the "toroidal" dynamics in the model as *g*_K1 _is varied using 1D recurrence maps. The 1D recurrence map defined as a plot of identified pairs, *V_n_*_+1 _versus *V_n_*, is shown in Figure 5a for the indicated values of *g*_K1 _for which a 2D-torus exists. In this map, an ergodic (or non-resonant) 2D-torus corresponds to an invariant circle. As long as the invariant circle remains smooth, the model exhibits quasi-periodic oscillations (Figure 2D). As the size of the torus becomes larger with increasing *g*_K1_, the invariant curve starts loosing smoothness that results in quick distortions of the torus shape. Further increase of *g*_K1 _leads to a resonance on the torus, corresponding to a stable periodic orbit comprised of a finite number of points, e.g., eight green dots in Figure 5a. This observation agrees well with a known scenario of torus breakdown [39,40]. In this scenario, the invariant circle becomes resonant with several periodic points emerging through a saddle-node bifurcation. The invariant circle becomes non-smooth when the unstable and stable manifolds of the saddle orbits start forming homoclinic tangles. Homoclinic tangles are well known to cause chaotic explosions in any system. In short, the breakdown of the non-smooth torus in the phase space is accompanied with the orchestrated onset of large-amplitude chaotic bursting. In terms of the map discussed here, the distorted invariant curve explodes into a chaotic attractor shown in Figure 5b. The middle part around -65 mV shows torus breaking, abruptly interrupted by the hyperpolarized passages in bursting corresponding to the left flat section of the map [41].

### Influence of other ionic currents

**Figure 6:**
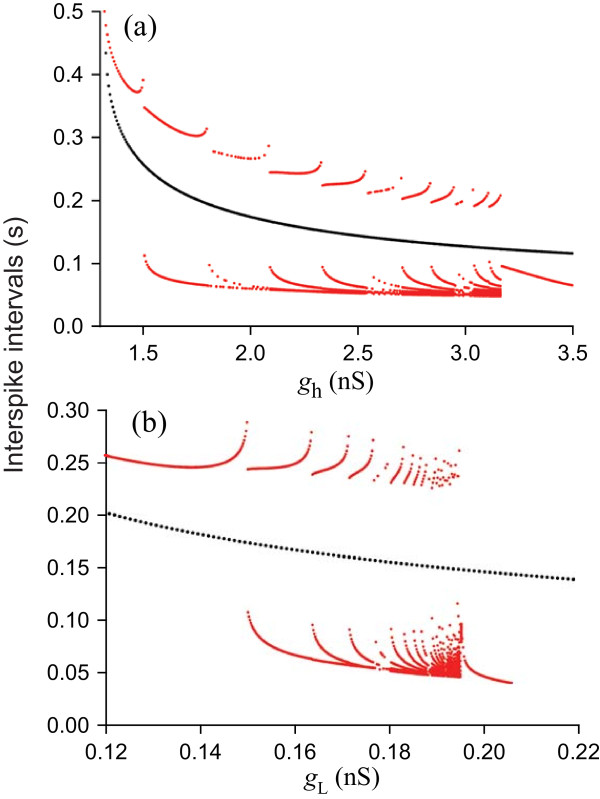
**Effect of the cation h-current conductance and the leak conductance on the dynamical regimes of the model**. Shown are bifurcation diagrams for the interspike intervals plotted versus *g*_h _**(a) **and *g*_L _**(b)**. On both panels red dots correspond to *g*_K1 _= 32 nS and *b *= 0.01; black dots correspond to *g*_K1 _= 32 nS and *b *= 0.1. Other parameters are the same as in Figure 2.

Variations of conductances of other ionic currents do not qualitatively reshape the oscillatory region bounded by the AH bifurcation curves in (*b*, *g*_K1_)-parameter plane, but lead to some quantitative shifts of the entire region (data not shown). Figure 6 exemplifies the influence of the cation h-current conductance and the leak conductance on the oscillatory regimes of the model. Both, the h-current and the leak current are inward, i.e., tend to depolarize the cell. We note that for the deterministic model the leak current is equivalent to the MET current. Thus, Figure 6b also shows the effect of the MET conductance on oscillatory regimes. When the parameters *b *and *g*_K1 _are set in the middle of the tonic oscillations region of Figure 2 (*b *= 0.1, *g*_K1 _= 32 nS), variations of *g*_h _and *g*_L _do not lead to any bifurcations of periodic tonic oscillations, but result in a gradual change of the oscillation period (black dots in Figure 6a,b). Small values of *g*_h _and *g*_L _result in slower- and larger-amplitude oscillations because of hyperpolarization of the cell and activation of the inward rectifier (K1) current. The increase of *g*_h _and *g*_L _results in faster oscillations terminated eventually at the depolarized equilibrium through the supercritical AH bifurcation. When the parameters of the model are poised in the center of the bursting region (*b *= 0.01, *g*_K1 _= 32 nS), variations of *g*_h _and *g*_L _result in a sequence of spike-adding bifurcations of bursting regimes (red dots in Figure 6a,b) similar to that shown in Figure 4c. Qualitatively, similar behavior was observed when the conductances of other ionic currents (DRK, Ca) were varied: i.e., gradual change of the period and amplitude of tonic oscillations or sequences of bifurcations of various tonic and bursting regimes when the model was poised, respectively, in the tonic oscillation or bursting region on the *b*-*g*_K1 _parameter plane.

To conclude, our results show that depending on the strength of outward BK currents, the model exhibits two distinct patterns of parameter dependence. For a relatively large strength of BK currents (*b >*0.02), the system is structurally stable within the oscillatory region, i.e., variations of the model parameters do not lead to qualitative transitions of oscillations. On the contrary, for small BK currents, *b <*0.02, the model passes through sequences of qualitative transitions generating a rich variety of periodic, quasi-periodic and chaotic oscillation patterns.

### Effect of the MET current fluctuations: stochastic dynamics

**Figure 7:**
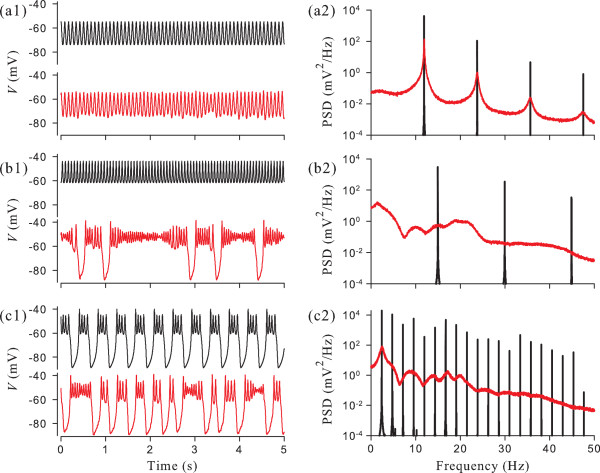
**Effect of thermal MET current fluctuations on spontaneous oscillations of the membrane potential**. Left column: voltage traces for the deterministic model with *ε *= 0 (black line, upper traces) and for the stochastic model with *ε *= 1 (red line, lower traces). (a1): *b *= 0.1, *g*_K1 _= 10 nS. (b1): *b *= 0.01, *g*_K1 _= 29 nS. (c1): *b *= 0.01, *g*_K1 _= 32 nS. Right column: PSDs corresponding to the voltage traces shown in (a1), (b1), and (c1). Other parameters: *g*_MET _= 0.65 nS, *g*_L _= 0.1 nS, *g*_Ca _= 1.2 nS, *g*_h _= 2.2 nS.

Thermal fluctuations of the MET current lead to two distinct effects on spontaneous oscillations of the membrane potential, depending on the strength of BK currents. For large values of the BK current strength, *b >*0.02, the MET noise leads to the well-known effect of amplitude and phase fluctuations of voltage oscillations [42], without changing the qualitative shape of oscillatory patterns (Figure 7a1). On the contrary, for smaller values of the BK conductance, *b <*0.02, noise leads to drastic qualitative changes in the membrane potential dynamics inducing complex burst-like activity (Figure 7b1, c1). These effects can also be characterized by the power spectral density (PSD). For large values of the BK conductance, fluctuations of the MET current merely lead to quantitative changes in the PSD: the delta peaks at the fundamental frequency of the oscillation and its higher harmonics get broadened by noise (Figure 7a2). In contrast, for smaller values of *b*, noise leads to qualitative changes in the PSD. Figure 7b provides an example for *b *= 0.01 and *g*_K1 _= 29 nS. In the absence of thermal noise, *ε *= 0, the model possesses a stable tonic periodic orbit. Thermal noise effectively shifts the model parameters toward the region of complex bursting oscillations, where fast, small-amplitude oscillations are interrupted sporadically by slow hyperpolarization excursions. The drastic effect of the thermal noise is best seen in the power spectrum, Figure 7b2: the narrow peak corresponding to the natural frequency of the deterministic oscillations is almost completely washed out. Furthermore, the coherence of fast oscillations is lost, presumably because of noise-induced hyperpolarization excursions, leading to a broadband PSD. The effect of noise on bursting is demonstrated in Figure 7c. The deterministic bursting orbit with four fast spikes per burst is characterized by a series of equidistant peaks in the PSD (Figure 7c2, black line), corresponding to the bursting frequency (2.18 Hz in this case) and its higher harmonics. Thermal fluctuations significantly alter the oscillation pattern and induce bursts, each with a random number of spikes (red line in Figure 7c1). This is similar to neuronal bursting models perturbed by noise [36]. The variability of the number of spikes in a burst is reflected in a broad peak at low frequency, corresponding to the bursting period and its higher harmonics. However, compared to noise-induced bursting in Figure 7b, the bursting intervals remain more regular. Consequently, the main peak at the frequency of bursting survives (Figure 7c2, red line).

### Noise-induced chaos

To better understand the origin of noise-induced variability of the membrane potential, we evaluated the largest Lyapunov exponent (LE) to measure the rate of separation of two solutions starting from close initial conditions in the phase space of the model. A stable equilibrium is characterized by a negative value of LE. Deterministic limit-cycle oscillations are characterized by a zero LE, indicating neutral stability of perturbations along the limit cycle. Positive values of the LE indicate irregular, i.e., chaotic oscillations [43]. In the case of a stochastic system, like the hair cell model with thermal noise, the LE can be interpreted in terms of convergence or divergence of responses of the system to repeated presentations of the same realization of noise [44]. A positive value of the LE indicates a chaotic irregular behavior whereby two trajectories of the model, which are subjected to identical noise and initially close to each other, diverge as time goes [45]. Oppositely, a negative value of the LE (two nearby trajectories converge on average) indicates insensitivity of the model to perturbations.

**Figure 8:**
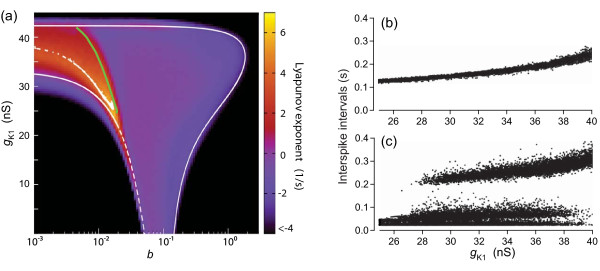
**The largest Lyapunov exponent (LE) and bifurcations of the noisy hair cell**. **(a) **The LE is shown as a function of the BK and K1 conductances *b *and *g*_K1_. White lines indicate the AH bifurcations of the deterministic system. The blue line demarcates the period doubling bifurcation of a large-amplitude limit-cycle (cf. Figure 2H,G). White dots indicate positive values of the LE for the deterministic system. Other parameters are the same as in Figure 7. **(b) **Interspike intervals vs the control parameter *g*_K1 _for *b *= 0.1. Black dots indicate instantaneous periods of the stochastic dynamics, i.e., taking into account a fluctuating MET current (*ε *= 1). **(c) **Interspike intervals versus *g_K_*_1 _for *b *= 0.01. Other parameters: *g*_MET _= 0.65 nS, *g*_L _= 0.1 nS, *g*_Ca _= 1.2 nS, *g*_h _= 2.2 nS.

The dependence of the LE on the two bifurcation parameters is shown as a color plot in Figure 8a. Regions of irregular and regular oscillations can be discerned. In the absence of noise, the LE is positive in an extremely narrow parameter region which is indicated by white dots in Figure 8a. One of these dots would correspond to the chaotic windows seen in Figure 4d. For relatively large values of the BK conductance, *b >*0.02, only regular oscillations are observed which are characterized by negative values of the LE. The LE becomes strongly negative beyond the region of deterministic oscillations bounded by the lines of the AH bifurcation. In the middle of this oscillation region, the LE is negative, but close to zero, indicating that limit-cycle oscillations are weakly affected by thermal noise. This is further demonstrated in a stochastic version of the bifurcation diagram (cf. Figure 4) of the interspike intervals in Figure 8b for *b *= 0.1. Although noise induces some variability of interspike intervals, no transition to bursting is observed throughout a range of *g*_K1_.

For small BK current strengths (*b <*0.02), a vigorous variability of the membrane potential is observed, characterized by large positive values of the LE. The region of noise-induced chaos with positive LE is singled out from that corresponding to large-amplitude tonic spiking by the boundary on which the first spike-adding bifurcation occurs (green line in Figure 8a). In this region, the deterministic model exhibits a plethora of distinct bursting patterns as the control parameters vary (see, e.g., Figure 4c). For example, for fixed values of *b *and *g*_K1 _within the bursting region, small variations of other parameter, e.g., leak conductance, *g*_L_, lead to a similar bifurcation transitions shown in Figure 6b. Noise enters the model equations through the MET conductance (2), and effectively modulates the leak conductance, *g*_L _+ *g*_MET _*P_o_*, where *P_o_*, the open probability of MET channels, fluctuates according to (3) and (4). For the parameter values used in Equation 3 and 4, the MET conductance *g*_MET _*P_o _*fluctuates within a range of 0.03-0.16 nS with the mean of 0.076 nS and with the standard deviation of 0.020 nS, sampling an interval of numerous bursting transitions shown in Figure 6b. Thus, the crucial effect of noise is that it induces sporadic transitions between structurally unstable bursting patterns. This is demonstrated by means of a plot of the interspike intervals for the stochastic system at *b *= 0.01 in Figure 8c: thermal noise wipes out all spike-adding bifurcations leading to a global variability of the instantaneous period of the membrane potential.

### Response to mechanical stimuli

The results of the preceding section showed three distinct regions of stochastic dynamics in the parameter space of the hair cell model: fluctuations around a stable equilibrium for parameters outside the oscillatory region; noisy limit-cycle oscillations for relatively large values of the BK conductance (*b >*0.02); and the region of irregular large-amplitude bursting oscillations for small values of *b*. In this section, we study how these distinct regimes of spontaneous stochastic dynamics affect tuning and amplification properties of the hair cell model in response to external mechanical stimuli.

### Sensitivity and frequency tuning

**Figure 9:**
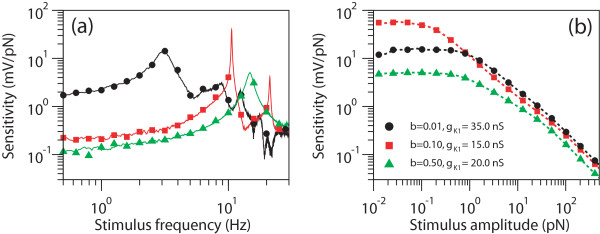
**Sensitivity of the hair cell membrane potential to an external mechanical force applied to the hair bundle**. **(a) **Sensitivity plotted as a function of stimulus frequency, i.e., frequency tuning curves, for values of *b *and *g*_K1 _as indicated. Symbols correspond to sensitivity values as computed using a sinusoidal stimulus with amplitude *F*_0 _= 0.1 pN. Solid lines correspond to a computation of the sensitivity on the basis of Equation 7 for a random Gaussian force that was band-limited to 200 Hz and had a standard deviation of *σ_s _*= 1 pN. **(b) **Sensitivity as a function of the amplitude of the sinusoidal external force. Values of *b *and *g*_K1 _as indicated. As stimulus frequency we used that of the respective maximal sensitivity in **(a) **(frequency of maximal linear response). Other parameters are the same as in Figure 8.

Note that an external force *F*_ext_(*t*) is included in the model through the mechanical compartment, see Equation 4. Figure 9 shows the sensitivity of the hair cell model to a sinusoidal external force, Equation 6. For parameter values outside the region of self-sustained oscillations, the sensitivity shows a broad small-amplitude peak at the frequency of noise-induced oscillations (Figure 9a, green line). In the parameter region of regular periodic oscillations, the hair cell model demonstrates a high sensitivity and selectivity to weak periodic force, characterized by a sharp peak at the natural frequency of self-sustained oscillations (Figure 9a, red line). Such a high selectivity is abolished by irregular complex oscillations for small values of the BK conductance. The typical frequency tuning curve in the region of the irregular oscillations (Figure 9a, black line) shows a broad peak at a low frequency and a sequence of smaller wide peaks at higher frequencies, corresponding to the fast voltage oscillations during bursts.

A conventional estimation of the frequency response is computationally expensive, as it requires variation of the frequency of an external sinusoidal force for a given set of parameters. In the regime of linear response, we used an alternative approach for estimation of sensitivity by stimulating the hair cell model with broadband Gaussian noise with small variance, so that the model operated in the linear response regime. This approach is widely used in neuroscience [28] and allows for accurate estimation of the sensitivity (Equation 7) at once for all frequencies within the band of the stimulating force. The cutoff frequency of the random stimulus was set to 200 Hz, i.e., much higher than natural frequencies of the model, so that random stimulus can be considered as white noise. Figure 9a shows that estimation of the sensitivity with sinusoidal and random stimuli gives very close results. Making use of such random stimuli, for a given parameter set of conductances *b *and *g_K_*_1_, we could therefore determine the best frequency eliciting a maximal response. For a driving at this best frequency, in Figure 9b we show the dependence of the sensitivity on the stimulus amplitude *F*_0_. The curve demonstrates a linear-response region for small *F*_0 _*<*1 pN, which is followed by a compressive nonlinearity. In the latter range, the sensitivity decays with the amplitude of the periodic force.

**Figure 10:**
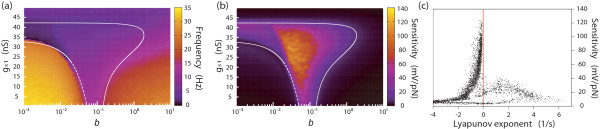
**Maximal sensitivity of the noisy hair cell**. Throughout the *b*-*g*_K1_-parameter plane, we determined the sensitivity of the stochastic model as a function of frequency. This was done using Equation 7 and a Gaussian external force that was band-limited to 200 Hz and had a standard deviation of 1 pN. For each choice of the parameters, we determined at which frequency the sensitivity was maximal. **(a) **Frequency map of the maximal sensitivity. As a color plot, we show the frequencies at which the sensitivity of the cell is maximal within the frequency band of the stimulus. **(b) **Maximum sensitivity map. Color coded, we plot the maximal sensitivity as a function of the bifurcation parameters *b *and *g_K_*_1_. In **(a, b)**, bifurcation lines of the AH bifurcation of the deterministic system are shown as white lines. **(c) **Scatter plot of the maximal sensitivity versus the LE of the model, both taken at the same values of the control parameters *b *and *g*_K1_. Note that the LE was determined in the absence of any external stimulus force but in the presence of noise. The vertical red line indicates where the LE is zero.

Turning back to the linear response of the model, we now inspect the sensitivity as a function of *b *and *g_K_*_1_. For each pair of parameters we recorded the maximal sensitivity over the entire frequency band of the stimulus and color coded this maximal sensitivity value and the value of the frequency corresponding to the maximal sensitivity. The result of these computations is shown in Figure 10a,b. First, we note that the region of noticeable sensitivity is bounded by the lines of the AH bifurcation, that is, the sensitivity of a spontaneously active hair cell is significantly higher than the sensitivity of a quiescent cell. Second, the region of maximal sensitivity is located in the center of the sensitivity map corresponding well to a region of the LE map where the LE is negative and close to zero. This is further illustrated in Figure 10c with a scatter plot of sensitivity versus the LE: sensitivity increases toward zero value of the LE. Small negative values of the LE refer to a longer transient time for perturbations to decay, which can be interpreted as higher values of an effective quality factor of oscillations. Consequently, in this region, the cell exhibits both high sensitivity and selectivity (narrow resonance peak) at frequencies of 5-15 Hz. The scatter plot in Figure 10c indicates also a second region where relatively high values of the sensitivity occur for positive values of the LE. In this region of irregular oscillations, the sensitivity is characterized by broad peaks at low frequencies (0-2 Hz) as exemplified in Figure 9a (black line) and shown in the sensitivity frequency map, Figure 10a. In this region of irregular oscillations, the hair cell model is not frequency selective, but possesses a high sensitivity to low-frequency or static stimuli.

### Response to static stimuli

**Figure 11:**
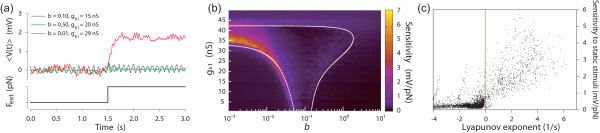
**Sensitivity of the hair cell model to static stimuli**. **(a) **Response of the membrane potential (upper panel) to a 0.5-pN step stimulus (lower panel) for values of the BK and K1 conductances as indicated. Other parameters as in Figure 7. **(b) **As a color plot, we show the static sensitivity as a function of the control parameters *b *and *g*_K1_. Static sensitivity was determined from frequency tuning curves (see Figure 10a) by averaging sensitivities in the frequency band 0.02-0.10 Hz. **(c) **Scatter plot of the static sensitivity versus the LE, both taken at the same values of the control parameters *b *and *g*_K1_. Note that the LE was determined in the absence of any external stimulus force but in the presence of noise. The vertical red line indicates where the LE is zero. AH bifurcation curves of the deterministic model are shown by white color in **(b)**.

The application of a step force stimulus illustrates the high sensitivity of the hair cell model to static stimuli in the regime of irregular oscillations (Figure 11a). Static variations of the MET current may shift the system to a different dynamical regime, e.g., with different burst patterns, leading to drastic changes in the averaged response. In the regular oscillations regime, a small variation of the MET current does not lead to a qualitative change in the oscillation pattern. Consequently, the response of the cell is weak (Figure 11a, black line), implying a weak sensitivity of the system to low-frequency stimuli. In the quiescent regime (Figure 11a, green line) the cell responds with damped oscillations typical for electrical resonance, again with small sensitivity. We determined the response to static stimuli from the frequency-dependent sensitivity by averaging over a frequency band from 0.02 to 0.1 Hz and show the result in Figure 11b as a map on the control parameter plane. Comparison with the map of the LE, Figure 8, indicates that the region of large values of static sensitivity and the region of large positive values of the LE correspond to each other. This is further demonstrated using the scatter plot in Figure 11c where the static sensitivity is plotted against the LE.

### Summary and conclusion

In this article, we investigated a Hodgkin-Huxley-type model that was developed to account for the spontaneous voltage oscillations observed in bullfrog saccular hair cells.

We determined its bifurcation structure in terms of two important conductances, associated with the inwardly rectifier (K1) and Ca^2+^-activated (BK) potassium currents. In the parameter space of the model, we isolated a region of self-sustained oscillations bounded by Andronov-Hopf bifurcation lines.

We found that for small values of BK and large values of K1 conductances the dynamics of the model is far more complicated than mere limit cycle oscillations, showing quasi-periodic oscillations, large-amplitude periodic spikes, and bursts of spikes. The model demonstrated a sequence of spike-adding transition similar to neuronal models belonging to a class of the so-called square-wave bursters [37,46]. However, the hair cell model also demonstrated a peculiar transition to bursting through quasi-periodic oscillations with two independent frequencies corresponding to a 2D torus in the phase space of the system. Specific mechanisms of the torus formation in detailed conductance-based models are not well studied, compared to mechanisms of torus dynamics in simplified models [33,47,48]. So far a canard-torus was reported recently in a model of cerebella Purkinje cells at a transition between tonic spiking and bursting regimes [49] with a mechanism related to a fold bifurcation of periodic orbits predicted in [50] and demonstrated in an elliptic burster model [33]. We showed that within small patches of parameter space at the transition from spiking to bursting and at the spike adding transition, voltage dynamics are chaotic, as evidenced by a positive LE.

Furthermore, we studied the effects of a noisy MET current on the statistics of the system. As a first step, we assessed the effects of such a stochastic input in the absence of any additional periodic stimulus. We showed that fluctuations can lead to drastic qualitative changes in the receptor potential dynamics. In particular, the voltage dynamics became chaotic in a wide area of parameter space. For a cell deep within the region of tonic oscillations, noise essentially resulted in a finite phase coherence of the oscillation.

To probe the possible role of voltage oscillations for signal processing by hair cells, we determined the response of the model to periodic mechanical stimulation of the noisy hair bundle. We found a high sensitivity and frequency selectivity for the regime of regular spontaneous oscillations. This result can readily be understood within the framework of periodically driven noisy nonlinear oscillators [51,52]. Hence, an oscillatory voltage compartment might constitute a biophysical implementation of a high-gain amplifier based on the physics of nonlinear oscillators.

Cells poised in the chaotic regime of low *b *and high *g_K_*_1 _respond well to low-frequency stimuli (*f <*3 Hz). In contrast, cells operating within the region of limit-cycle oscillations (high *b *and moderate *g_K_*_1_) possess a pronounced frequency selectivity with a high best frequency (*f >*5 Hz).

We found that the transition between these two response regimes roughly occurs at the boundary separating the chaotic regime with positive Lyapunov exponent from the regime of perturbed tonic oscillations associated with purely negative Lyapunov exponents. Note that the latter boundary was defined for the noisy system in the absence of periodic stimulation. Moreover, in both regimes we uncovered strong correlations between the sensitivity and the Lyapunov exponent, whereas in the regime of tonic oscillations the sensitivity is strongest for negative but small exponents, in the chaotic regime there was an approximately linear correlation between sensitivity and positive Lyapunov exponent. These remarkable findings should be further explored. In particular, it would be desirable to clarify whether simpler models that are capable to show chaos as well as limit-cycle oscillations display similar correlations between sensitivity and Lyapunov exponents in these different regimes.

Next to tonic voltage oscillations, also irregular bursting of hair cells has experimentally been observed [16]. Within the framework of the employed model, these qualitatively different dynamics can faithfully be reproduced, suggesting a parameter variability among saccular hair cells. Our results show that these different dynamical regimes are associated with quite distinct response properties with respect to mechanical stimulation. Important stimuli for the sacculus of the bullfrog are seismic waves with spectral power mainly at higher frequencies and quasi-static head movements predominantly at low frequencies [53]. Our results suggest that the observed variability in hair cell voltage dynamics could have functional significance, reflecting a differentiation of hair cells into distinct groups specialized to sensory input of disparate frequency content.

Another possible role of spontaneous voltage oscillations could be in the regularization of stochastic hair bundle oscillations via the phenomenon of reverse electro-mechanical transduction [17]. Recently, it has been argued on theoretical grounds that oscillations of the membrane potential may synchronize stochastic hair bundle oscillations, thus improving frequency selectivity and sensitivity of the mechanical compartment of the hair cell [19,20]. Moreover, an experimental study has documented that basolateral ionic currents indeed have a significant effect on the statistics of stochastic hair bundle oscillations [54]. For example, it has been observed that the pharmacological blockage of BK currents leads to more regular hair bundle oscillations of lower frequency. Our results suggest that this may be due to a shift of the working point of the voltage compartment into the region of self-sustained voltage oscillations. When operating in this regime, high-quality voltage oscillations entraining the hair bundle could effectively reduce its stochasticity. Besides coupling-induced noise reduction in groups of hair bundles [55,56], this mechanism could thus constitute an alternative way to diminish the detrimental effect of fluctuations in hair cells.

In summary, this study established the electrical oscillator found in saccular hair as an oscillatory module capable of nonlinear amplification. This further supports the idea of nonlinear oscillators playing a crucial role in the operation of the inner ear. The interplay between different oscillatory modules (active hair bundle motility and electric oscillations) remains to be explored in more detail in future investigations.

## Competing interests

The authors declare that they have no competing interests.

## Authors' contributions

AS, LH and AN carried out simulation and bifurcation analysis of the deterministic model. AN carried out calculation of the Lyapunov exponent. KD and BL carried out numerical calculations of the sensitivity. AN, KD, BL and AS wrote the paper. All authors read and approved the final manuscript. 

## Appendix: description of ionic currents

**The inwardly rectifier current **(*I*_K1_) [14] is modeled by a combination of one fast and one slow activation gates,

(8) $$\begin{array}{c} I_{\text{K}1}=g_{\text{K}1}\left(V-E_{\text{K}}\right)\;\left[0.7m_{\text{Klf}}\left(V\right)+0.3\;m_{\text{Kls}}\left(V\right)\right],\\ \tau_{\text{Klf},\text{s}}\frac{dm_{\text{K}1\text{f},\text{s}}}{dt}=m_{\text{K}1\infty }-m_{\text{Klf},\text{s}},\;\;m_{\text{K}1\infty }={\left[1+\exp\;\left(\left(V+110\right)∕11\right)\right]}^{-1},\\ \tau_{\text{Klf}}=0.7\exp\;\left[-\left(V+120\right)∕43.8\right]+0.04,\;\;\tau_{\text{Kls}}=14.1\exp\;\left[-\left(V+120\right)∕28\right]+0.04, \end{array}$$

with *E*_K _= -95 mV and the maximum conductance *g*_K1 _used as the control parameter in the model.

**Cation h-current **(*I*_h_) [14,57] is modeled with three independent activation gates,

(9) $$\begin{array}{c} I_{\text{h}}=g_{\text{h}}\;\left(V-E_{\text{h}}\right)\;\left[3m_{\text{h}}^{2}\left(1-m_{\text{h}}\right)+m_{\text{h}}^{3}\right],\\ \tau_{\text{h}}\frac{dm_{\text{h}}}{dt}=m_{\text{h}\infty }-m_{\text{h}},\;\;m_{\text{h}\infty }={\left[1+\exp\;\left(\left(V+87\right)∕16.7\right)\right]}^{-1},\\ \tau_{\text{h}}=63.7+135.7\exp\;\left[-{\left(\frac{V+91.4}{21.2}\right)}^{2}\right], \end{array}$$

with the maximum conductance *g*_h _= 2.2 nS and *E*_h _= *-*45 mV.

**The DRK current **(*I*_DRK_) [14] is modeled with two independent activation gates and is given by the Goldman-Hodgkin-Katz (GHK) current equation,

(10) $$\begin{array}{c} I_{\text{DRK}}=P_{\text{DRK}}\frac{VF^{2}}{RT}\frac{{\left[\text{K}\right]}_{\text{in}}-{\left[\text{K}\right]}_{\text{ex}}e^{-FV∕RT}}{1-e^{-FV∕RT}}m_{\text{DRK}}^{2},\\ \tau_{\text{DRK}}\frac{dm_{\text{DRK}}}{dt}=m_{\text{DRK}\infty }-m_{\text{DRK}},\;m_{\text{DRK}\infty }={\left[1+\exp\;\left(\left(V+48.3\right)∕4.19\right)\right]}^{-1∕2},\\ \tau_{\text{DRK}}={\left(\alpha_{\text{DRK}}+\beta_{\text{DRK}}\right)}^{-1},\;\alpha_{\text{DRK}}={\left(3.2e^{-V∕20.9}+3\right)}^{-1},\;\beta_{\text{DRK}}={\left(1467e^{V∕5.96}+9\right)}^{-1}, \end{array}$$

where *P*_DRK _= 2.4 × 10^-14 ^L/s is the maximum permeability of *I*_DRK_; [K]_in _= 112 mM and [K]_ex _= 2 mM are intracellular and extracellular K^+ ^concentration; *F *and *R *are Faraday and universal gas constants; *T *= 295.15 K is the temperature.

**Voltage-gated Ca**^2+ ^**current **(*I*_Ca_) is modeled with three independent gates [58,59],

(11) $$\begin{array}{c} I_{\text{Ca}}=g_{\text{Ca}}m_{\text{Ca}}^{3}\left(V-E_{\text{Ca}}\right),\\ \tau_{\text{Ca}}\frac{dm_{\text{Ca}}}{dt}=m_{\text{Ca}\infty }-m_{\text{Ca}},\;m_{\text{Ca}\infty }={\left[1+\exp\;\left(-\left(V+55\right)∕12.2\right)\right]}^{-1},\\ \tau_{\text{Ca}}=0.046+0.325\exp\;\left[-{\left(\frac{V+77}{51.67}\right)}^{2}\right], \end{array}$$

where *g*_Ca _= 1.2 nS is the maximum Ca^2+ ^conductance and *E*_Ca _= 42.5 mV.

**Ca**^2+^**-activated potassium currents **(BKS and BKT) [14] were modeled with the GHK current equation. The kinetic scheme of both BK currents was the same as in the Hudspeth-Lewis model [58] with three closed (*C*_0_, *C*_1_, *C*_2_) and two open (*O*_2_, *O*_3_) states. The open probability of BK channel is *O*_2 _+ *O*_3_. The transient BK channel (BKT) has an additional inactivation gate, characterized by the gating variable *h*_BKT _so that the BK currents are given by

(12) $$I_{\text{BKS}}=b\;P_{\text{BKS}}\frac{VF^{2}}{RT}\frac{{\left[\text{K}\right]}_{\text{in}}-{\left[\text{K}\right]}_{\text{ex}}e^{-FV∕RT}}{1-e^{-FV∕RT}}\;\left(O_{2}+O_{3}\right),$$

(13) $$I_{\text{BKT}}=b\;P_{\text{BKT}}\frac{VF^{2}}{RT}\frac{{\left[\text{K}\right]}_{\text{in}}-{\left[\text{K}\right]}_{\text{ex}}e^{-FV∕RT}}{1-e^{-FV∕RT}}\;\left(O_{2}+O_{3}\right)\;h_{\text{BKT}},$$

where *P*_BKS _= 2 *× *10^-13 ^L/s and *P*_BKS _= 14 *× *10^-13 ^L/s are maximal permeabilities while the dimensionless quantity *b *parameterizes the strength of these currents and was used as the control parameter of the model. The kinetics of Ca-activated BK currents is given by

(14) $$\begin{array}{c} \frac{dC_{1}}{dt}=k_{1}\left[\text{Ca}\right]\;C_{0}+k_{-2}C_{1}-\left(k_{-1}+k_{2}\left[\text{Ca}\right]\right)C_{1},\\ \frac{dC_{2}}{dt}=k_{2}\left[\text{Ca}\right]\;C_{1}+\alpha_{c}O_{2}-\left(k_{-2}+\beta_{c}\right)\;C_{2},\\ \frac{dO_{2}}{dt}=\beta_{c}C_{2}+k_{-3}O_{3}-\left(\alpha_{c}+k_{3}\left[\text{Ca}\right]\right)O_{2},\\ \frac{dO_{3}}{dt}=k_{3}\left[\text{Ca}\right]\;O_{2}-k_{-3}O_{3},\;C_{0}=1-\left(C_{1}+C_{2}+O_{2}+O_{3}\right), \end{array}$$

and complemented by the dynamics of the Ca^2+ ^concentration, [Ca] [14],

(15) $$\frac{d\left[\text{Ca}\right]}{dt}=-0.00061\;I_{\text{Ca}}-2800\;\left[\text{Ca}\right].$$

The parameters in Equation 14 were the same as in the Hudspeth-Lewis model (see Table two in [58]) except for *β_c _*which in our simulation was *β_c _*= 2500 s*^-^*^1 ^similar to the model used in [16].

Finally, the voltage-gated inactivation for transient BK channels is [14]

(16) $$\begin{array}{c} \tau_{\text{BKT}}\frac{dh_{\text{BKT}}}{dt}=h_{\text{BKT}\infty }-h_{\text{BKT}},\;h_{\text{BKT}\infty }={\left[1+\exp\;\left(\left(V+61.6\right)∕3.65\right)\right]}^{-1},\\ \tau_{\text{BKT}}=2.1+9.4\exp\;\left[-\;{\left(\left(V+66.9\right)∕17.7\right)}^{2}\right]. \end{array}$$

**The leak current **(*I*_L_) is given by

(17) $$I_{\text{L}}=g_{\text{L}}\left(V-E_{\text{L}}\right),$$

where *g*_L _is the leak conductance and *E*_L _= 0 mV.

## Endnote

^a^While the two-parameter bifurcation diagram in Figure 2 was obtained using parameter continuation software CONTENT and MATCONT [24,25], the one-parameter bifurcation diagram for the interspike intervals was obtained by direct numerical simulation of the deterministic model: for each *g*_K1 _value the model equations were numerically solved for a total time interval of 20s; the sequence of interspike intervals was collected and plotted against *g*_K1_.
